# Supplementary material for: Microglia are not protective against cryptococcal meningitis
Source: Nat Commun. 2023 Nov 8;14:7202. doi: 10.1038/s41467-023-43061-0 (PMC10632471; doi:10.1038/s41467-023-43061-0)
Supplement: Supplementary file 1 — Supplementary Information [file 41467_2023_43061_MOESM1_ESM.pdf]

Supplementary Figures

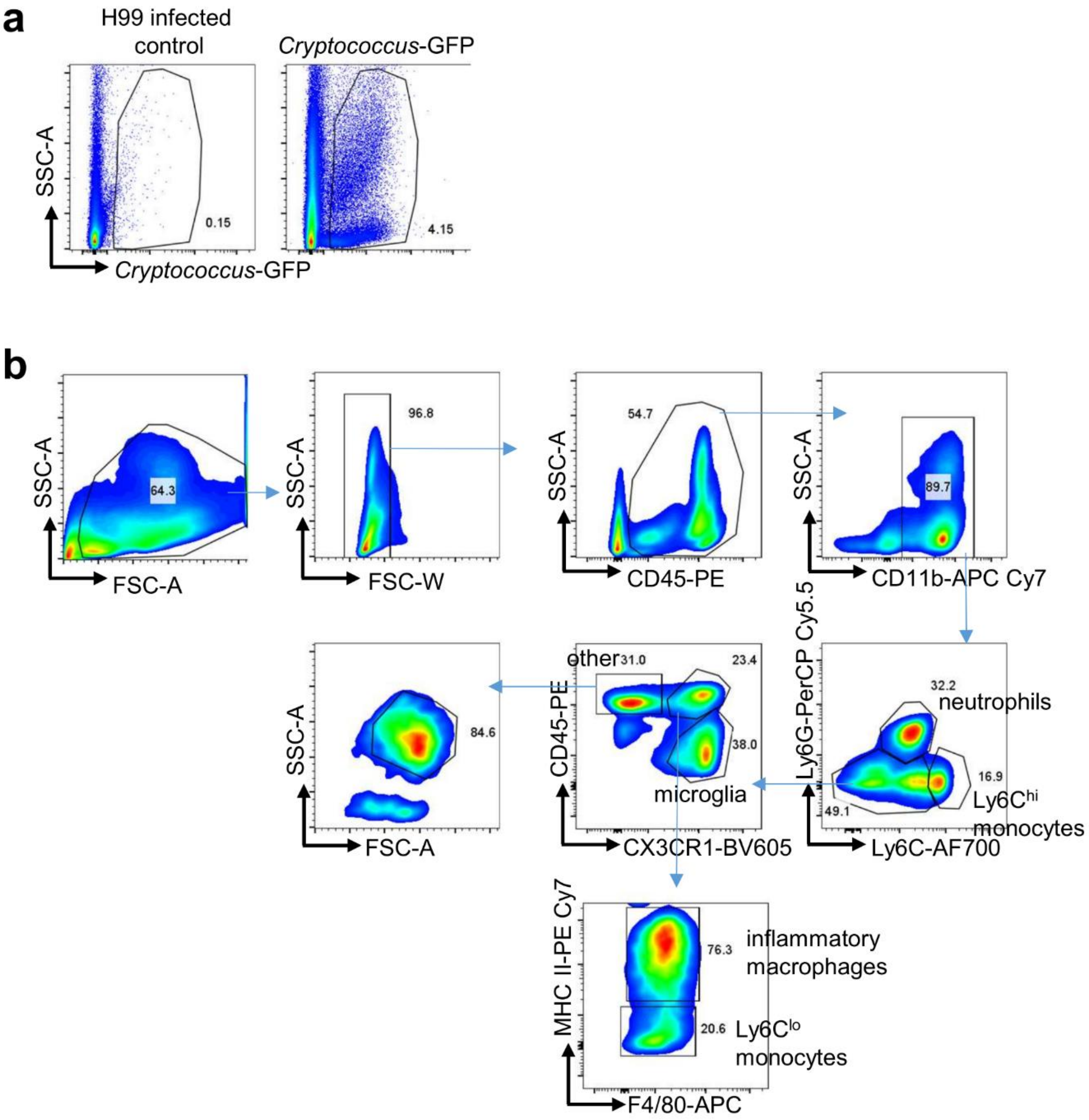

Figure S1. Gating strategies used to analyse (**a**) yeast population or (**b**) host leukocytes in the infected mouse brain. In (a), plots are ungated and show examples from mice infected with non-fluorescent control H99 (as a gating control) and GFP-expressing C. neoformans. In (b), the main immune cell populations routinely gated on in our experiments are shown. Within the 'other' gate, we found that most cells are SSC^{high} and are likely eosinophils.

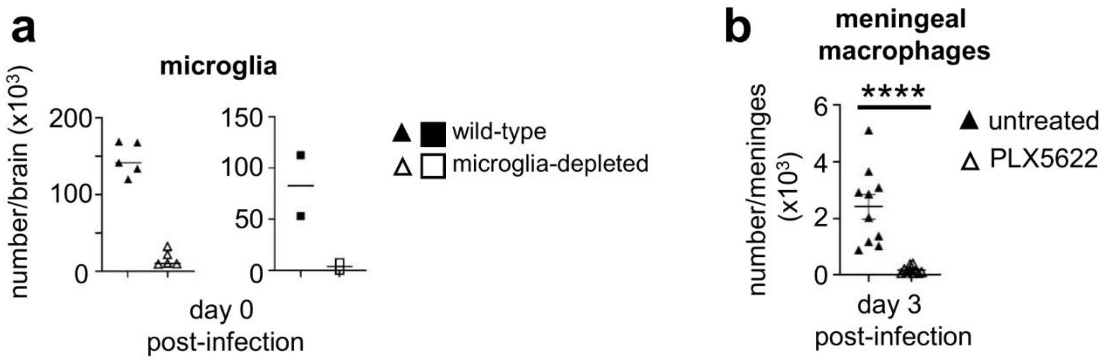

Figure S2. (a) Microglia depletion efficiency in uninfected mice using either PLX5622 (left graph, triangle symbols) or *Sall1*^{CreER}*Csf1r*^{flox} mice (right graph, square symbols). Data shown with the mean and is from one experiment, each point represents an individual mouse. **(b)** Number of meningeal macrophages in untreated (n=10) and PLX5622-treated (n=10) C57BL/6 mice at day 3 post-infection. Data shown with mean +/- SEM and is pooled from 2 independent experiments and analysed by unpaired two-tailed t-test. ****P<0.0001

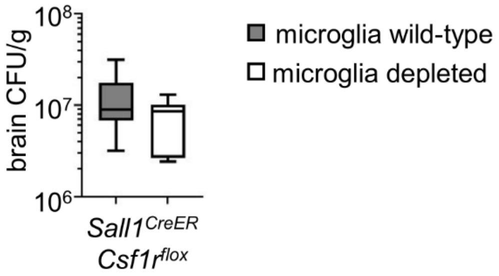

Figure S3. Fungal brain burdens at day 6 post-infection in wild-type (Cre-) and microglia-depleted (Cre+) animals (n=8 mice per group). Data pooled from 2 independent experiments. Box plots show median with 25%/75% percentiles and maximum and minimum values.

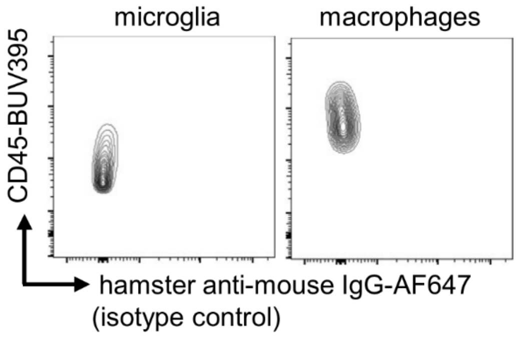

Figure S4. Intracellular staining of microglia and macrophages with isotype control antibody (to accompany CXCL9 staining shown in Fig 6).
