# Supplementary material for: Microglia are not protective against cryptococcal meningitis
Source: Nat Commun. 2023 Nov 8;14:7202. doi: 10.1038/s41467-023-43061-0 (PMC10632471; doi:10.1038/s41467-023-43061-0)
Supplement: Supplementary file 3 — Reporting Summary [file 41467_2023_43061_MOESM3_ESM.pdf]

nature portfolio

Corresponding author(s): Rebecca Drummond

Last updated by author(s): 16/10/2023

Reporting Summary

Nature Portfolio wishes to improve the reproducibility of the work that we publish. This form provides structure for consistency and transparency in reporting. For further information on Nature Portfolio policies, see our Editorial Policies and the Editorial Policy Checklist.

Statistics

For all statistical analyses, confirm that the following items are present in the figure legend, table legend, main text, or Methods section.

n/a	Confirmed	
☐	☒	The exact sample size (*n*) for each experimental group/condition, given as a discrete number and unit of measurement
☐	☒	A statement on whether measurements were taken from distinct samples or whether the same sample was measured repeatedly
☐	☒	The statistical test(s) used AND whether they are one- or two-sided<br>*Only common tests should be described solely by name; describe more complex techniques in the Methods section.*
☒	☐	A description of all covariates tested
☐	☒	A description of any assumptions or corrections, such as tests of normality and adjustment for multiple comparisons
☐	☒	A full description of the statistical parameters including central tendency (e.g. means) or other basic estimates (e.g. regression coefficient) AND variation (e.g. standard deviation) or associated estimates of uncertainty (e.g. confidence intervals)
☐	☒	For null hypothesis testing, the test statistic (e.g. *F*, *t*, *r*) with confidence intervals, effect sizes, degrees of freedom and *P* value noted<br>*Give P values as exact values whenever suitable.*
☒	☐	For Bayesian analysis, information on the choice of priors and Markov chain Monte Carlo settings
☒	☐	For hierarchical and complex designs, identification of the appropriate level for tests and full reporting of outcomes
☒	☐	Estimates of effect sizes (e.g. Cohen's *d*, Pearson's *r*), indicating how they were calculated

Our web collection on statistics for biologists contains articles on many of the points above.

Software and code

Policy information about availability of computer code

Data collection	BD FACS Diva v9.0, ZEN Black 3.1, ISX
Data analysis	FlowJo v10.6.1, GraphPad Prism 9, ZEN Blue 3.1, IDEAS v6.2

For manuscripts utilizing custom algorithms or software that are central to the research but not yet described in published literature, software must be made available to editors and reviewers. We strongly encourage code deposition in a community repository (e.g. GitHub). See the Nature Portfolio guidelines for submitting code & software for further information.

Data

Policy information about availability of data

All manuscripts must include a data availability statement. This statement should provide the following information, where applicable:

- Accession codes, unique identifiers, or web links for publicly available datasets
- A description of any restrictions on data availability
- For clinical datasets or third party data, please ensure that the statement adheres to our policy

All raw data associated with the figures is available on request, without restrictions, and is provided in the source data file.

Research involving human participants, their data, or biological material

Policy information about studies with human participants or human data. See also policy information about sex, gender (identity/presentation), and sexual orientation and race, ethnicity and racism.

Reporting on sex and gender	N/A
Reporting on race, ethnicity, or other socially relevant groupings	N/A
Population characteristics	N/A
Recruitment	N/A
Ethics oversight	N/A

Note that full information on the approval of the study protocol must also be provided in the manuscript.

Field-specific reporting

Please select the one below that is the best fit for your research. If you are not sure, read the appropriate sections before making your selection.

☒ Life sciences ☐ Behavioural & social sciences ☐ Ecological, evolutionary & environmental sciences

For a reference copy of the document with all sections, see nature.com/documents/nr-reporting-summary-flat.pdf

Life sciences study design

All studies must disclose on these points even when the disclosure is negative.

Sample size	In each of the animal experiments, we aimed to have n=6 per experiment group, since our power calculations indicated that this sample size would allow for a detection of a 30% difference in the means with a probability of greater than 95%, assuming a standard deviation of around 19% and minimum power 0.8. These calculations are based on years of experience with these models and measuring these parameters using our chosen methods. When the desired sample size was not available (due to mouse breeding and availability), we used what was available and repeated the experiment several times independently to increase sample size number closer to the desired number.<br>The same power calculations were applied to determine sample size for the cell line-based experiments.
Data exclusions	No data exclusions.
Replication	All experiments were repeated at least twice and up to 4 times, to ensure reproducibility of the results. Data from each experiment was pooled and raw data is presented in the figures. In cases where the experiment-to-experiment variability was higher, data was normalized to the appropriate control and pooled data presented in this way, after ensuring that individual experiments had a similar pattern of results and gave similar statistical results as the pooled normalized data. There were no experiments that we could not replicate independently in this study.
Randomization	In all experiments, the assignment of littermate controls to cages, the placement of the experimental cage within the animal facility and the order in which infections/analysis procedures were performed were done at random.
Blinding	In some experiments, mouse blinding was not possible due to the nature of mouse breeding. For example, when heterozygous crosses were used to generate littermates, genotyping had to be performed to identify appropriate animals and assess group sizes prior to the experiment taking place. In some of the experiments that used littermate controls (e.g. Sall1-CreER-Csf1r-flox animals - see methods for details), animals were assigned an ID number during genotyping (and experiment designed on this basis) before being analyzed in a blinded fashion using the ID number. Genotype was assigned at the final analysis stage (i.e. when graphing the data). All other data were acquired and analyzed in a non-blinded fashion because it did not involve subjective measurements.

Reporting for specific materials, systems and methods

We require information from authors about some types of materials, experimental systems and methods used in many studies. Here, indicate whether each material, system or method listed is relevant to your study. If you are not sure if a list item applies to your research, read the appropriate section before selecting a response.

Materials & experimental systems

n/a	Involved in the study	
☐	☒	Antibodies
☐	☒	Eukaryotic cell lines
☒	☐	Palaeontology and archaeology
☐	☒	Animals and other organisms
☒	☐	Clinical data
☒	☐	Dual use research of concern
☒	☐	Plants

Methods

n/a	Involved in the study	
☒	☐	ChIP-seq
☐	☒	Flow cytometry
☒	☐	MRI-based neuroimaging

Antibodies

Antibodies used	Anti-mouse antibodies used in this study were: CD45 (30-F11), CD11b (M1/70), CX3CR1 (SA011F11), MHC Class II (M5/114.15.2), F480 (BM8), Ly6G (1A8), Ly6C (HK1.4), CXCL9 (MIG-2F5.5) all from Biolegend. These antibodies were used at 1:200 final dilution. For microscopy experiments, the following antibodies were used: Iba-1 antibody (Wako), Alexa-Fluor 647 secondary antibody (Thermofisher). These antibodies were used at 1:500 final dilution.
Validation	All antibodies against surface-expressed markers used in this study have been previously validated by the manufacturer, as stated on their associated product webpages, and by our own lab in previous experiments.

Eukaryotic cell lines

Policy information about cell lines and Sex and Gender in Research

Cell line source(s)	BV-2 microglia were obtained from Dr Michail Lionakis (National Institutes of Health). BV-2 were originally made by Dr Elizabeth Blasi and are deposited in the Biological Bank of the IRCCS San Martino University Hospital (Italy).
Authentication	Expression of common microglia markers CD45, CD11b and Cx3CR1 by BV-2 microglia were tested by flow cytometry.
Mycoplasma contamination	Cell lines were not tested for mycoplasma; all cells grew well and health/viability was checked prior to each experiment.
Commonly misidentified lines (See ICLAC register)	No misidentified/contaminated cell lines used in this study.

Animals and other research organisms

Policy information about studies involving animals; ARRIVE guidelines recommended for reporting animal research, and Sex and Gender in Research

Laboratory animals	Sex-matched mice were used at 8-12 weeks of age, using either male or female mice depending on availability. Wild-type in this study refers to C57BL/6 (Charles River) or the corresponding littermates of genetically-modified lines; Cx3cr1-CreERRosa26iDTR, Sall1-CreERRosa26Ai14, and Sall1-CreERCsf1rflox. Cx3cr1-CreER, Rosa26iDTR, Rosa26Ai14 and Csf1rflox mice were originally purchased from Jackson and colonies bred and maintained at the University of Birmingham. Sall1-CreER mice were a kind gift from Dr Melanie Greter (University of Zurich).
Wild animals	No wild animals were used in the study.
Reporting on sex	Experiments with transgenic mice utilised both males and females to maintain littermate controls. We used female mice for experiments with wild-type mice only, since female mice can be housed in larger groups/smaller cage numbers.
Field-collected samples	No field collected samples were used in the study.
Ethics oversight	Animal studies were approved by the Animal Welfare and Ethical Review Board at the University of Birmingham and UK Home Office under Project Licence PBE275C33.

Note that full information on the approval of the study protocol must also be provided in the manuscript.

Flow Cytometry

Plots

Confirm that:

☐ The axis labels state the marker and fluorochrome used (e.g. CD4-FITC).

☒ The axis scales are clearly visible. Include numbers along axes only for bottom left plot of group (a 'group' is an analysis of identical markers).

☒ All plots are contour plots with outliers or pseudocolor plots.

☒ A numerical value for number of cells or percentage (with statistics) is provided.

Methodology

Sample preparation	Isolated leukocytes were resuspended in PBS and stained with Live/Dead stain (Invitrogen) on ice as per manufacturer's instructions. Fc receptors were blocked with anti-CD16/32 and staining with fluorochrome-labelled antibodies was performed on ice. Labelled samples were acquired immediately or fixed in 2% paraformaldehyde prior to acquisition.
Instrument	Samples were acquired on a 5 laser BD LSR Fortessa.
Software	The flow cytometer software used to collect the data was BD FACSDiva, and the final analysis was completed using FlowJo (TreeStar).
Cell population abundance	The abundance of myeloid populations analysed in this study are shown in Fig 3 and the gating strategy in Fig S2. Post-sort analyses confirmed the purity of our sorted populations as >95%.
Gating strategy	Our gating strategy is shown in Fig S1.

☒ Tick this box to confirm that a figure exemplifying the gating strategy is provided in the Supplementary Information.
